# Asia Cohort Consortium: Challenges for Collaborative Research

**DOI:** 10.2188/jea.JE20120024

**Published:** 2012-07-05

**Authors:** Minkyo Song, Betsy Rolland, John D. Potter, Daehee Kang

**Affiliations:** 1 Department of Biomedical Sciences, Seoul National University College of Medicine, Seoul, Korea; 2 Cancer Prevention Program, Division of Public Health Sciences, Fred Hutchinson Cancer Research Center, Seattle, Washington, United States; 3 Department of Human Centered Design & Engineering, College of Engineering, University of Washington, Seattle, Washington, United States; 4 Department of Preventive Medicine, Seoul National University College of Medicine, Seoul, Korea

**Keywords:** Asia, cohort, consortium

## Abstract

In this era of chronic diseases, large studies are essential in investigating genes, environment, and gene–environment interactions as disease causes, particularly when associations are important but not strong. Moreover, to allow expansion and generalization of the results, studies should be conducted in populations outside Western countries. Here, we briefly describe the Asia Cohort Consortium (ACC), a collaborative cancer cohort research project that was first proposed in 2004 and now involves more than 1 million healthy individuals across Asia. There are approximately 50 active members from Bangladesh, China, India, Japan, Korea, Malaysia, Singapore, Taiwan, Thailand, the United States, and elsewhere. To date, the work of the ACC includes 3 articles published in 2011 on the roles of body mass index, tobacco smoking, and alcohol consumption in mortality, diabetes, and cancer of the small intestine. Many challenges remain, including data harmonization, resolution of ethical and legal issues, establishment of protocols for biologic samples and transfer agreements, and funding procurement.

## INTRODUCTION

Prospective cohort studies provide the best level of observational evidence on disease causation. Furthermore, prospective cohort studies have specific strengths over clinical trials, which are often regarded as more powerful than observational studies in the hierarchy of evidence. For instance, in situations where it is unethical to design an experimental study (eg, in situations involving exposure to tobacco, alcohol, or obesity), observational studies are the only way to undertake research. Further, unlike clinical trials, cohort studies can assess multiple outcomes for any 1 exposure or multiple exposures for a specific outcome. Chronic diseases are on the rise worldwide, and Asian countries face a growing disease burden and the many important health challenges that result.^1^ The patterns and causes of diseases may differ among populations, yet much remains unknown about the causes of diseases in Asians, who account for most of the global population.^2^^,^^3^ Approximately 95% of genome-wide association studies have been undertaken among people of European origin. Thus, the findings may not apply to other populations and the resulting genomic medicine might benefit only the few. Hence, a wider spectrum of populations should be investigated.^4^

Since the completion of the Human Genome Project, epidemiologic studies encompassing genetics have prospered, and the importance of prospective cohorts has been more widely recognized.^5^ Moreover, a sufficiently large cohort or a population laboratory is essential for understanding the roles of genetic variation, environmental exposures, and the interaction between genes and exposures in the development of a disease.^6^ Analyses of gene–environment interactions in complex diseases with small interaction odds ratios or of genome–environment-wide interactions require even larger sample sizes to confirm associations.^7^^,^^8^ Potter has suggested that a cohort of at least 1 000 000 ethnically diverse individuals (“the Last Cohort”) is essential to discover disease susceptibility, early-detection biomarkers, and more-precise phenotypes.^9^

Combining existing cohorts is a strategy to achieve the desired results more quickly, more cheaply, and with similar scientific validity.^10^^,^^11^ Consortia are being established in and across many nations and research groups in order to produce expedited results and better understand the extent of international ethnic variation.^10^^,^^12^ One example is the Cohort Consortium of the National Cancer Institute (NCI), a collaboration of 43 high-quality cohorts consisting of more than 4 million people.^13^ The Cohort Consortium includes 4 signature initiatives that are actively producing valuable results: the Body Mass Index (BMI) and All Cause Mortality Pooling Project; the Breast and Prostate Cancer Cohort Consortium (BPC3); the Pancreatic Cancer Cohort Consortium; and the Vitamin D Pooling Project (VDPP). Through such collaborations, the complex process of identifying disease causes in relation to various genetic determining factors can be approached systematically.

## HISTORY AND ORGANIZATION OF THE ACC

The Asia Cohort Consortium (ACC) is another large consortium of cohort-based studies in Pacific Rim countries, with approximately 50 active members from China, India, Bangladesh, Japan, Korea, Malaysia, Singapore, Taiwan, Thailand, the United States, and elsewhere (Table 1).^14^^,^^15^ The ACC was first proposed in November 2004 at a meeting in Seoul, Korea, and nearly all researchers at the meeting agreed on the need to establish a large consortium of cohorts. By establishing a cohort of at least 1 million healthy individuals around the world who will be followed until various disease endpoints, the ACC seeks to identify associations of diseases with genetics, environmental exposures, and their interaction, and to discover early-detection biomarkers.^16^ The ACC aims “(i) to serve as a platform for cross-cohort collaborative projects and combined analysis and (ii) to act as an incubator for new cohorts.” Led by co-chairs John Potter of the Fred Hutchinson Research Center, USA and Daehee Kang, Department of Preventive Medicine, Seoul National University College of Medicine, Korea, the ACC investigators meet biannually to update progress on existing and new cohorts in each country, to share ideas on data harmonization and development of common protocols, and to prepare collaborative projects. The ACC Coordinating Center (ACC CC) is located at the Fred Hutchinson Cancer Research Center and offers support for scientific collaboration, operations management, statistical and data management, and communications infrastructure and tool development. The activities of the ACC CC are described in detail elsewhere.^14^

**Table 1.** Summary of members of the Asia Cohort Consortium

Country	Cohort	No. of subjects	Date of enrollment	Mean follow-up period	Mean age at entry
India					
	Mumbai Cohort Study	146 820	1991–1997	5.2	50.8
	Trivandrum Oral Cancer Screening Trial (TOCS)	129 097	1995–2002	7.5	49.5
Bangladesh					
	Health Effects of Arsenic Longitudinal Study	11 452	2000–2002	6.6	37.1
Mainland China					
	China National Hypertension Survey Epidemiology Follow-up Study (CHEFS)	154 737	1990–1992	7.2	55.4
	Shanghai Cohort Study (SCS)	18 100	1986–1989	16.3	55.3
	Shanghai Men′s Health Study (SMHS)	61 379	2001–2006	3.1	54.9
	Shanghai Women′s Health Study (SWHS)	74 873	1996–2000	8.6	52.1
	Linxian Cohort	29 459	1984–1987	18.5	51.9
Taiwan					
	Community-based Cancer Screening Project (CBCSP)	23 763	1991–1992	15.2	47.3
	Cardiovascular Disease Risk Factor Two-Township Study (CVCFACTS)	5 129	1990–1993	14.9	47.0
Singapore					
	Singapore Chinese Health Study (SCHS)	63 242	1993–1999	11.5	56.5
Japan					
	Three-Prefecture Cohort Study, Aichi (3-Pref Aichi)	32 210	1985	11.6	56.2
	Ibaraki Prefectural Health Study	97 578	1993–1994	11.5	58.8
	Japan Collaborative Cohort Study (JACC)	86 671	1988–1990	12.7	57.6
	Japan Public Health Center-based Prospective Study 1 (JPHC1)	42 771	1990–1992	14.4	49.6
	Japan Public Health Center-based Prospective Study 2 (JPHC2)	55 712	1992–1995	11.5	54.2
	Three-Prefecture Cohort Study, Miyagi (3-Pref Miyagi)	29 525	1984	11.6	56.9
	Miyagi Cohort Study	44 867	1990	12.8	52.0
	Ohsaki National Health Insurance Cohort Study	47 670	1995	9.9	60.1
Korea					
	Korea Multi-center Cancer Cohort (KMCC)	16 013	1993–2004	6.5	55.6
	Seoul Male Cohort	13 953	1992–1993	14.7	49.2

The second meeting of the ACC was held in April 2005 at the Fred Hutchinson Cancer Research Center. At that meeting, the Steering Committee was established, consisting of principal investigators from various cohort studies in each participating country. To address and resolve the issues of standardization and harmonization across different cohort studies, Working Groups were established in the areas of diet and nutrition, obesity and physical activity, occupation and environment, alcohol and tobacco use, family history and genetics, biospecimens and sample collection, data collection and management, and follow-up and endpoint ascertainment. Currently, consortium members are collaborating on projects examining: body mass index (BMI) and various outcomes; rare cancers, including cancers of the small intestine and pancreas, which are infrequent and thus difficult to study in single studies; and biospecimen use across existing Asian cohorts.

Proposals initiating a collaborative project can be submitted throughout the year. Before every biannual meeting, all proposals are reviewed by the Executive Committee (EC) and ACC members. Proposals from anyone interested in collaborating with the ACC are also reviewed, including those from non-ACC scientists who are sponsored by an ACC member. Data analysis and project coordination are performed by the Coordinating Center at the Fred Hutchinson Cancer Research Center.

## PROGRESS AND RESULTS

ACC participants are self-funded, and individual projects usually require a grant proposal to secure funding. The ACC has 2 funded grant proposals from the US NCI on rare cancers and BMI and on BMI and mortality, as well as other projects under review. Larger grant proposals are currently being drafted.

The first cross-cohort collaborative project, BMI in Asian populations, has yielded 3 articles to date (Table 2).^17^^–^^19^ The first article published by the group was a pooled analysis of BMI and risk of death among more than 1.1 million persons from 19 cohorts in Asia.^17^ We reported a U-shaped association between BMI and mortality in East Asians that subtly differed from the associations observed in studies of North American and European populations. These observations were possible largely because, in Asia, we were able to study the association between very low BMI and mortality, an opportunity that has not existed in populations of European origin for many decades. A U-shaped association was also reported in a 2011 pooled analysis of 7 large-scale cohort studies in Japan.^20^ In contrast, among Indians and Bangladeshis, there was no elevated risk of death in high BMI groups. These results provide important new public health data, as few previous studies have been conducted on BMI and overall risk of death in Asian populations, which account for more than 60% of the world’s population. The second article was a pooled cross-sectional analysis of BMI and self-reported diabetes that included more than 900 000 individuals from 18 cohorts.^18^ As was the case for BMI and mortality, the overall impact of BMI on diabetes risk had not been adequately studied in an Asian population. This study encompassed 7 Asian countries and established the shape and strength of the association between BMI and diabetes. Similar to the previous results, a positive association between BMI and diabetes prevalence was shown in all analyzed cohorts and all subgroups of the study population. The third, and most recent article, published by the ACC reported the findings of a study of the association of cancer of small intestine with BMI, tobacco smoking, and alcohol drinking, using a pooled analysis of over 500 000 individuals from 12 cohorts.^19^ The analysis of 134 incident cases showed a trend toward an increased hazard ratio of 1.50 (95% CI, 0.76–2.96) in the high BMI (BMI >27.5 kg/m^2^) group. Although the results were not statistically significant, they support the hypothesis that elevated BMI is a risk factor for cancer of the small intestine. No association was observed for tobacco smoking or alcohol drinking.

**Table 2.** Publications of the Asia Cohort Consortium

No. of individuals included in analysis	No. of cohorts used in analysis	Exposure	Outcome	Main result^d^	Ref
1 141 609	19	BMI	Total mortality and cause- specific mortality	BMI ≤15.0 HR = 2.8 (1.9–4.1)^a^ BMI >35.0 HR = 1.5 (1.3–1.7)^a^	17

934 154	18	BMI	Self-reported diabetes	BMI <15.0 OR = 0.6 (0.3–0.8)^b^ BMI ≥35.0 OR = 2.2 (1.9–2.7)^b^	18

527 726	12	BMI, tobacco smoking, alcohol drinking	Incidence of cancer of small intestine	BMI >27.5 HR = 1.5 (0.8–3.0)^c^	19

Abbreviation: BMI, body mass index; HR, hazard ratio; OR, odds ratio.

Reference BMI categories: ^a^22.6–25.0, ^b^22.5–24.9, ^c^22.6–25.0.

^d^95% CI.

## FUTURE PLANS

Many challenges remain for the ACC. The first is harmonizing data from legacy cohorts. For instance, some questions on exposures were asked differently and demand more careful standardization. Even more complex is the need for new infrastructure, such as the establishment of an Asian nutrient database. Much work is left to be done in the field of nutrition, especially when using data from multiple countries.^21^^,^^22^ Furthermore, standardizing and harmonizing new and continuing cohorts, and incorporating them into the existing study in a valid way, is similarly complex and time-consuming. Phenotypic measures, specific measurement tools, and other areas may be discordant across studies.^11^ Biospecimens present unique challenges. The quality of samples collected and stored under different protocols and for varying periods may differ among cohorts. Sample transfer between countries is sometimes a problem. Ethical and legal issues regarding informed consent of study participants and the use of their information for international studies are other areas that need to be resolved. To fulfill the collective enthusiasm and talent of workers in the ACC, funding is a constant necessity. We have begun well, but there is a great deal left to do.
